# Effect of intravitreal C_3_F_8_ gas in patients with vitreomacular traction: A retrospective case series

**DOI:** 10.1007/s00717-017-0382-5

**Published:** 2017-12-11

**Authors:** Anna-Maria Haas, Christoph Mayer, Anton Haas, Werner Wackernagel

**Affiliations:** Department of Ophthalmology, Medical University of Graz, Auenbruggerplatz 4, 8036 Graz, Austria

**Keywords:** Intravitreal gas, Perfluoropropane (C3F8), Pneumatic vitreolysis, Vitreomacular adhesion, Vitreomacular traction, Intravitreales Gas, Perfluoropropan (C_3_F_8_), Pneumatische Vitreolyse, Vitreomakuläre Adhäsion, Vitreomakuläre Traktion

## Abstract

**Background:**

We aimed to assess the efficacy of a single intravitreal perfluoropropane (C_3_F_8_) gas injection for the treatment of vitreomacular traction with or without a macular hole.

**Methods:**

In this retrospective case series, seven eyes of six patients with symptomatic vitreomacular traction documented on optical coherence tomography, one with a macular hole additionally, received a single intravitreal C_3_F_8_ gas injection of up to 0.3 ml. The primary endpoint was vitreomacular traction release at 1 month after injection. Secondary endpoints included resolution of vitreomacular adhesion within 6 months, nonsurgical closure of macular holes, and change in central foveal thickness and best-corrected visual acuity.

**Results:**

Overall, on optical coherence tomography, six of seven eyes (85.7%) had release of vitreomacular traction during the entire study duration: three within 1 month of injection and the other three within 6 months. Of the latter group, two of the three eyes showed a concurrent epiretinal membrane and one concurrent diabetic retino- and maculopathy. The patient with a macular hole had resolution of vitreomacular traction within 1 month but had to undergo vitrectomy because of nonclosure of the macular hole. Associated adverse events were macular edema with a consequent lamellar hole after injection in one patient, and another patient developed retinal detachment.

**Conclusion:**

Intravitreal C_3_F_8_gas injection is an inexpensive and promising minimally invasive option for the treatment of symptomatic and persistent vitreomacular traction with or without a macular hole. Further larger studies, especially comparing C_3_F_8_ gas injection with other treatment options, are needed.

## Zusammenfassung

**Hintergrund:**

Ziel der vorliegenden Fallserie war es, die Wirksamkeit einer einmaligen intravitrealen Applikation von Perfluoropropangas (C_3_F_8_) für die Behandlung der vitreomakulären Traktion mit oder ohne Makulaloch zu beurteilen.

**Methoden:**

In dieser retrospektiven Fallserie bekamen 7 Augen von 6 Patienten mit einer symptomatischen vitreomakulären Traktion in der optischen Kohärenztomographie, eine davon mit zusätzlich einem Makulaloch, eine einmalige intravitreale C_3_F_8_-Gasinjektion von bis zu 0,3 ml. Der primäre Endpunkt war die Lösung der vitreomakulären Traktion einen Monat nach der Injektion. Die sekundären Endpunkte beinhalteten die Lösung der vitreomakulären Traktion innerhalb von 6 Monaten, die Verschließung eines Makulalochs ohne weitere vitreoretinale Intervention, Veränderungen in der zentralen fovealen Dicke und der Sehschärfe.

**Ergebnisse:**

Insgesamt bestand bei 6 von 7 Augen (85,7 %) eine mit optischer Kohärenztomographie dokumentierte Lösung der vitreomakulären Traktion; 3 innerhalb eines Monats und 3 weitere innerhalb eines halben Jahres. Von den Letzteren wiesen 2 der 3 Augen gleichzeitig eine epiretinale Membran auf und eines eine simultane diabetische Retino- und Makulopathie. Der Patient mit einem Makulaloch zeigte innerhalb eines Monats eine vitreomakuläre Traktionslösung, musste sich jedoch wegen Persistenz des Makulalochs einer Vitrektromie unterziehen. Assoziierte unerwünschte Ereignisse waren ein Makulaödem mit einem Schichtloch nach der Injektion bei einem Patienten und eine Netzhautablösung bei einem anderen Patienten.

**Schlussfolgerung:**

Die intravitreale Gasinjektion mit C_3_F_8_-Gas ist eine kostengünstige und vielversprechende minimalinvasive Option für die Behandlung von symptomatischer vitreomakulärer Traktion mit oder ohne Makulaloch. Weitere größere Studien, in der v. a. die C_3_F_8_-Gasinjektion mit anderen Therapieoptionen verglichen wird, sind erforderlich.

## Introduction

Posterior vitreous detachment, typically occurring between the ages of 45 and 65 years, is defined as the separation of the vitreous body from the internal limiting membrane of the retina and is a physiological age-related process [1]. When the vitreous fails to detach completely, vitreomacular adhesion (VMA), showing no retinal abnormalities, or vitreomacular traction (VMT), with detectable retinal changes on optical coherence tomography, is the consequence. Typical symptoms of VMT are decreased vision and metamorphopsia [2].

Current treatment options include observation, when patients are either asymptomatic or when symptoms do not aggravate, or medical therapy with ocriplasmin and pars plana vitrectomy, which remains the mainstay of treatment when there is no indication for ocriplasmin or the treatment fails. Data from the Microplasmin for Intravitreal Injection–Traction Release Without Surgical Treatment (MIVI-TRUST) trial showed the nonsurgical success of VMT release within 28 days in 41.7% of cases, which was statistically significant, and closure of the macular hole (MH) in 30% [3].

Three studies, conducted by Rodrigues et al., Steinle et al., and Chan et al., investigating the release rate of VMT and MH closure using intravitreal perfluoropropane (C_3_F_8_) gas showed very promising results [4–6]. The purpose of the present case series was to further examine the effect of a single intravitreal C_3_F_8_ gas injection with patients showing symptomatic VMT with or without MH.

## Materials and methods

A retrospective case series of patients who elected to undergo an intravitreal C_3_F_8_ injection for the treatment of symptomatic VMT syndrome, including when associated with MH, between March 2013 and September 2015 was performed at the Department of Ophthalmology at the Medical University of Graz. In total, seven eyes of six patients, with a mean age of 60.4 years (range, 43–75 years), were treated with a C_3_F_8_ gas injection. All patients underwent baseline testing of Snellen visual acuity, biomicroscopy of the anterior and posterior segment, and spectral domain optical coherence tomography (SD-OCT). This case series was in accordance with the Declaration of Helsinki, and the approval of the ethics committee of the Medical University of Graz was obtained.

All intravitreal injections of expansile gas were carried out in an operating room and were in all cases performed by the same surgeon. Following the use of topical 1% Tetracaine anesthetic, the patients were prepared for surgery using an eyelid speculum, drape, and lavage of the conjunctival sac with povidone-iodine; subsequently, up to 0.3 ml of 100% C_3_F_8_ gas was injected. Three eyes received 0.2 ml and four eyes 0.3 ml of 100% C_3_F_8_ gas. Using a 30-gauge needle on a 1-ml tuberculin syringe, the gas was injected through the pars plana at a distance of 3.5 mm from the limbus. In all patients, an anterior chamber paracentesis was performed.

After surgery, the patients were told to avoid supine positioning because of the risk of cataract formation. Postoperatively, the patients had to use ofloxacin eye drops three times daily for 4 days. The patients were followed up for 1–2 weeks after gas injection and subsequently at least monthly until release of VMT.

The primary outcome measure was the release rate of VMT validated using SD-OCT at 1 month. Secondary measurements of interest included release of VMT within 6 months, change in central foveal thickness and in best-corrected VA, and closure of the MH. Only a change of best corrected visual acuity (BCVA) of two or more Snellen lines was regarded as significant.

To be included in this case series, the patients had to present with an SD-OCT-confirmed VMT with an adherence diameter of less than 1500 μm.

Exclusion criteria were patients who had undergone a vitrectomy or intravitreal injection before or who had an active ocular infection, an age-related macular degeneration or a glaucoma, an operative ocular intervention less than 3 months earlier, or presented with a retinal detachment in the other eye. Informed consent from all patients was obtained for inclusion in the study.

## Results

Seven eyes of six patients underwent an intravitreal 100% C_3_F_8_ gas injection for the treatment of VMT and MH. Three of the patients were male and three female; both eyes of one woman were assessed. The patient demographics as well as the pre- and posttreatment characteristics are listed in Table 1.

**Table 1:** Pretreatment and posttreatment patient characteristics

								Before treatment			After treatment				
Case	Age	Sex	Eye	Diagnosis	Lens status	ERM	DM	VA	CFT	EOA	VA	CFT	PPV	Adverse events	Outcome
1	66	M	OD	VMT	P	Yes	Yes	0.125	780	367	0.1	571	No	LH	QS
2	66	M	OS	VMT	P	Yes	No	0.25	590	803	0.4	816	Yes	Retinal tear and detachment	FAIL
3	43	F	OD	VMT	P	No	No	0.63	446	217	0.8	200	No	None	AS
4	72	M	OS	VMT with small MH	P	No	No	0.1	–	218	0.2	–	Yes	Nonclosure of MH	AS
5	50	F	OS	VMT	P	No	No	1.0	335	397	1.0	205	No	None	AS
6	51	F	OD	VMT	P	No	No	0.5	351	229	1.0	204	No	None	QS
7	75	F	OD	VMT	P	Yes	No	0.5	603	69	0.5	270	No	None	QS

*AS* absolute success (defined as VMT release within 1 month of treatment), *CFT* central foveal thickness (in μm), *DM* diabetes mellitus, *EOA* extend of adhesion (in μm), *ERM* epiretinal membrane, *F* female, *FAIL* failure (defined as no VMT release), *LH* lamellar hole, *M* male, *MH* macular hole, *OD* right eye, *OS* left eye, *P* phakic, *PPV* pars plana vitrectomy, *QS* qualified success (defined as VMT release later than 1 month after injection), *VA* visual acuity (in logMAR units), *VMT* vitreomacular traction

Three of the seven eyes (42.86%) had concurrent epiretinal membranes (ERM), among which one (14.26%) presented with concurrent diabetic retino- and maculopathy. Mean time from diagnosis to operation was 63 days (range, 6–223 days; SD = 75.86) and mean adhesion diameter was 328.57 μm (range, 63–803 μm; SD = 235.66). The patient with the extent of adhesion of 803 μm did not have VMT release. Traction release by 1 month after injection was observed in three of the seven eyes (42.86%), while a further three eyes detached within 6 months (85.71% final release rate). In two of the three eyes with concurrent ERM there was VMT release; however, not within 1 month but after 5 and 10 weeks. Furthermore, in one of one eye with concurrent diabetic retino- and maculopathy there was release within 10 weeks. One of the six patients had a small MH (201 μm) with VMT. Although the VMT released successfully after 1 week the MH did not close and hence underwent vitrectomy. The average number of days until VMT resolution was 54 (range, 7–173 days; median 28). Visual acuity improved by two or more Snellen lines in 42.86% and remained stable in 57.14% of cases.

Overall, the mean central foveal thickness decreased in patients with release of VMT from 517.5 μm (range, 335–780 μm; SD = 171.82) to 377.67 μm (range, 200–816; SD = 257.93) before and after treatment, respectively.

One patient showed macula edema with a consequent lamellar hole after the injection, whereas another patient developed a retinal tear with a retinal detachment with subsequent vitrectomy. No patient sustained complications during the intravitreal gas injection.

## Case presentations

### Patient 1

**Fig. 1:**
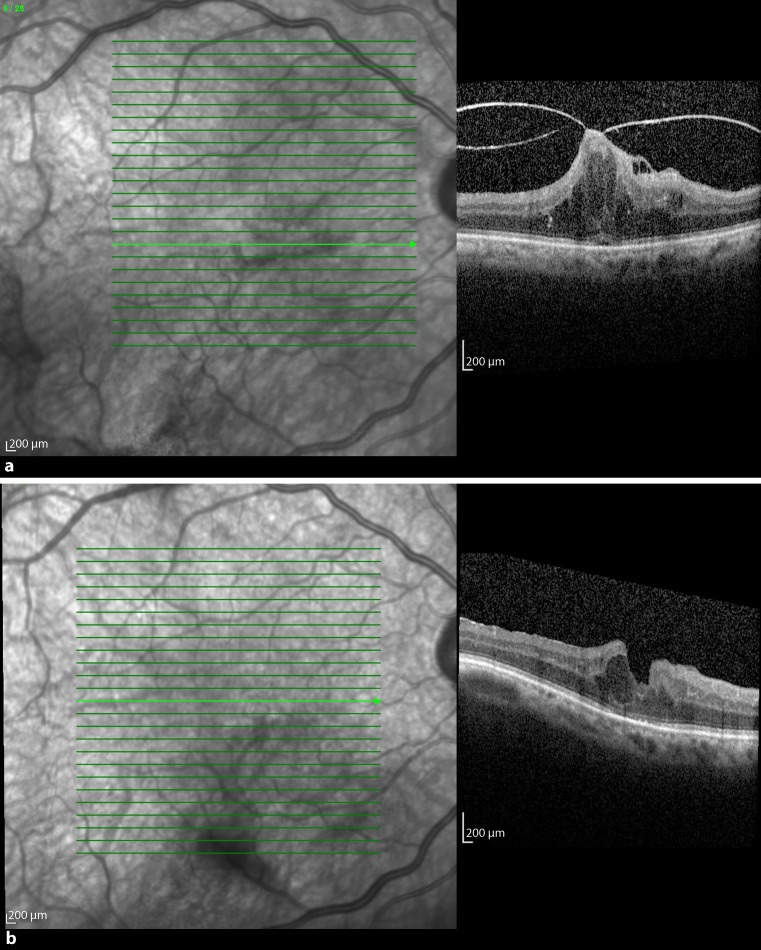
**a** Optical coherence tomography images of patient 1 obtained just before C_3_F_8_ intravitreal injection. The figure shows a vitreomacular traction with schisis-like splitting of the retina. The posterior vitreous is still attached at the optic disc. **b** Ten weeks after injection the vitreomacular traction was released, but a lamellar hole with intraretinal cysts developed

Patient 1 was a 66-year-old man. He presented with an SD-OCT-confirmed VMT in the right eye in March 2013 (Fig. 1a). Secondary ophthalmological findings were diabetic maculopathy, proliferative diabetic retinopathy, as well as a macula pucker. The preoperative BCVA was 0.125, the adhesion diameter, 367 μm, and foveal thickness, 780 μm. The patient was observed for 7 months (223 days), before he was treated with 0.3 ml C_3_F_8_ gas. Three weeks after the intravitreal injection, no release of traction was observed, but he had decreased vision due to a macular edema. VMT released 10 weeks after the intervention, with the macular edema persisting. The foveal thickness was 571 μm after treatment and BCVA after resolution of traction was 0.1 (Fig. 1b). There was no further reduction of the diabetic macular edema, and therefore the patient received two injections of bevacizumab intravitreally 9 months after gas application.

### Patient 2

**Fig. 2:**
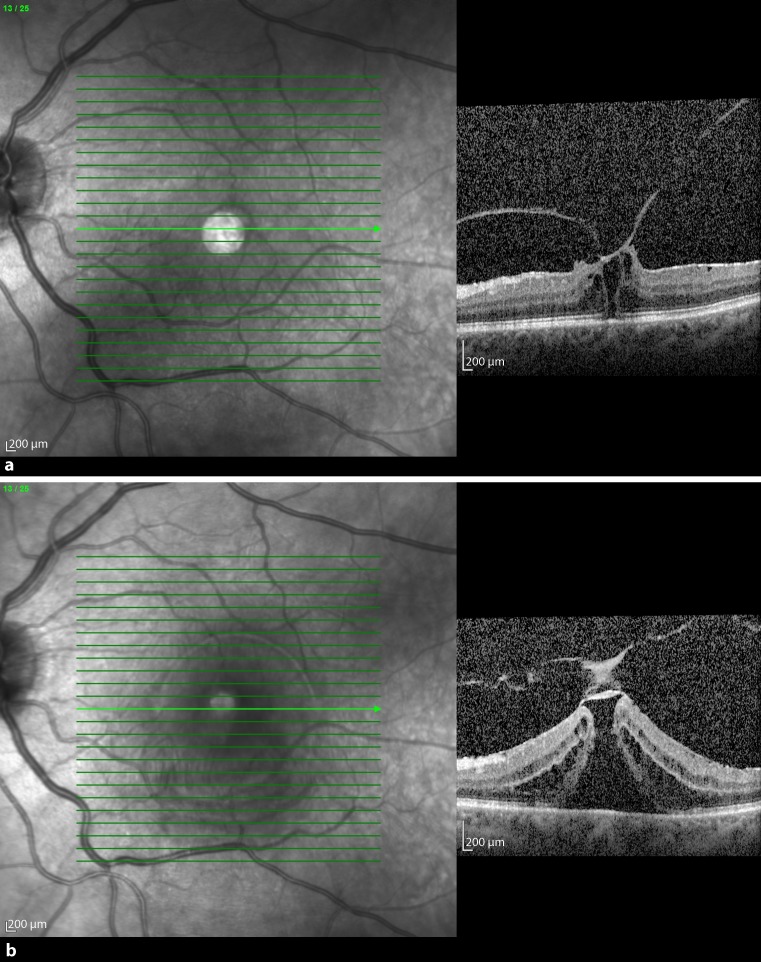
**a** Baseline spectral domain optical coherence tomography (OCT) of patient 2 with vitreomacular traction. OCT showed that the maximal diameter of the adhesion in horizontal scans was 803 μm. The retinal anatomy was disrupted under the adhesion zone. **b** Three weeks after the injection, no release of VMT was seen and the retinal thickness increased by 236 µm

The second patient was a 66-year-old man who presented in August 2013 with a VMT in the left eye diagnosed with SD-OCT (Fig. 2a). Additionally, he had an ERM on OCT. After 2 months (84 days) of watchful waiting the BCVA decreased to 0.25, foveal thickness was 580 μm, and the extent of adhesion was 803 μm. The patient underwent an intravitreal injection of 0.3 ml 100% C_3_F_8_ gas. One week after the procedure, there was no release of traction. At the 3‑week follow-up after the procedure, the patient complained of a black shadow. While BCVA increased to 0.4, no release of traction was seen and the foveal thickness increased to 816 μm (Fig. 2b). In the periphery at the 6 o’clock position there was a retinal tear with retinal detachment, which implied vitrectomy with gas. After resorption of the gas, the retina stayed attached with the same visual outcome.

### Patient 3

**Fig. 3:**
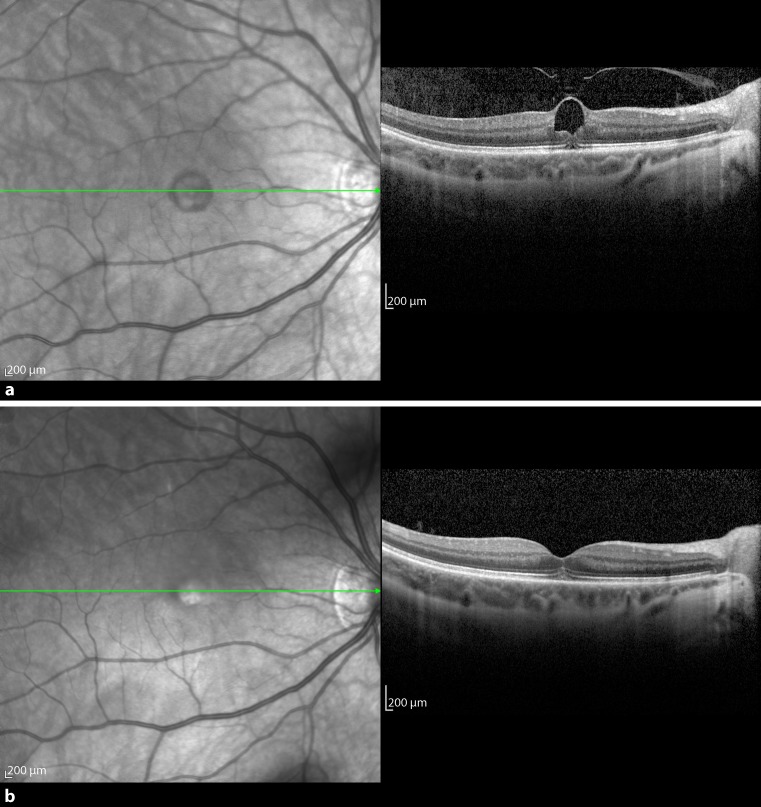
**a** Preoperative optical coherence tomography of patient 3 with vitreomacular traction (VMT) and a huge cyst in the inner retinal layer. The ellipsoid zone underneath the VMT was disrupted. **b** VMT release and restoration of the ellipsoid zone 12 days postoperatively

Patient 3 was a 43-year-old woman who presented with metamorphopsia and decrease of central vision in her right eye in October 2013. Focal VMT was diagnosed on OCT with an adhesion diameter of 217 μm, a foveal thickness of 446 μm, and a BCVA of 0.63 (Fig. 3a). Six days later she received an intravitreal injection of 0.2 ml C_3_F_8_ gas. The vitreous released successfully 12 days after the injection with a postoperative foveal thickness of 200 μm and an improvement of BCVA to 0.8 (Fig. 3b).

### Patient 4

**Fig. 4:**
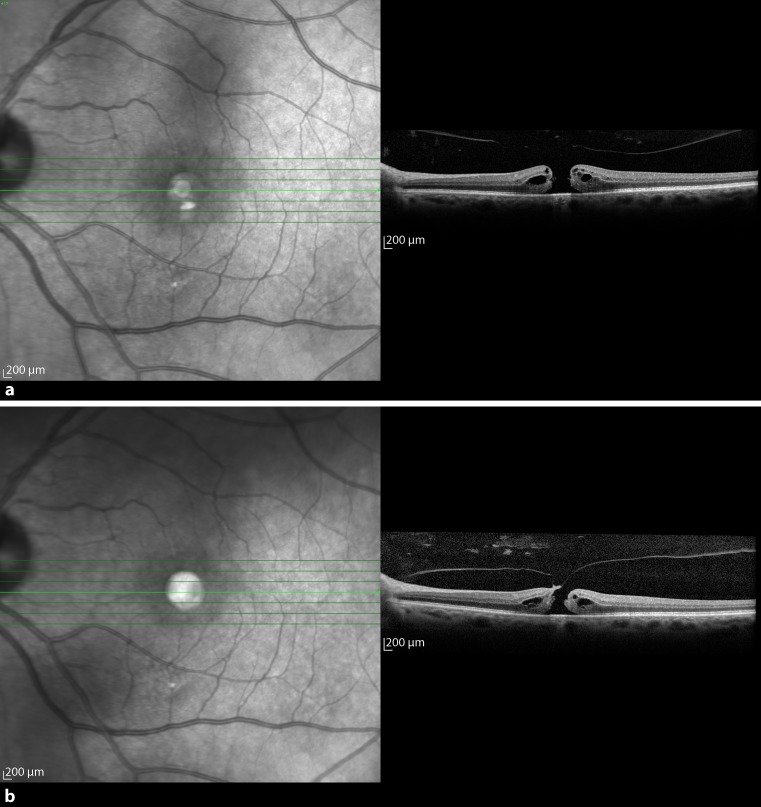
**a** Baseline spectral domain optical coherence tomography of patient 4 with a macular hole and vitreomacular traction (VMT). **b** One week after injection of gas, the VMT released but the macular hole did not close. Therefore, 5 weeks later the patient underwent vitrectomy with gas

The fourth patient, a 72-year-old man, saw a blind spot in the central vision in his left eye in October 2013, which was diagnosed on SD-OCT as a small MH (201 μm) with an adhesion expanse of 218 μm (Fig. 4a). Initially he had a BCVA of 0.1. One week later, 0.2 ml C_3_F_8_ gas was injected into the vitreous. After another 1 week, there were no more signs of tractional forces seen on OCT; however, the hole remained open and increased to 475 μm (Fig. 4b). Since the MH failed to close after 1.5 months, successful vitrectomy with gas was performed with an increase of BCVA to 0.2.

### Patient 5

**Fig. 5:**
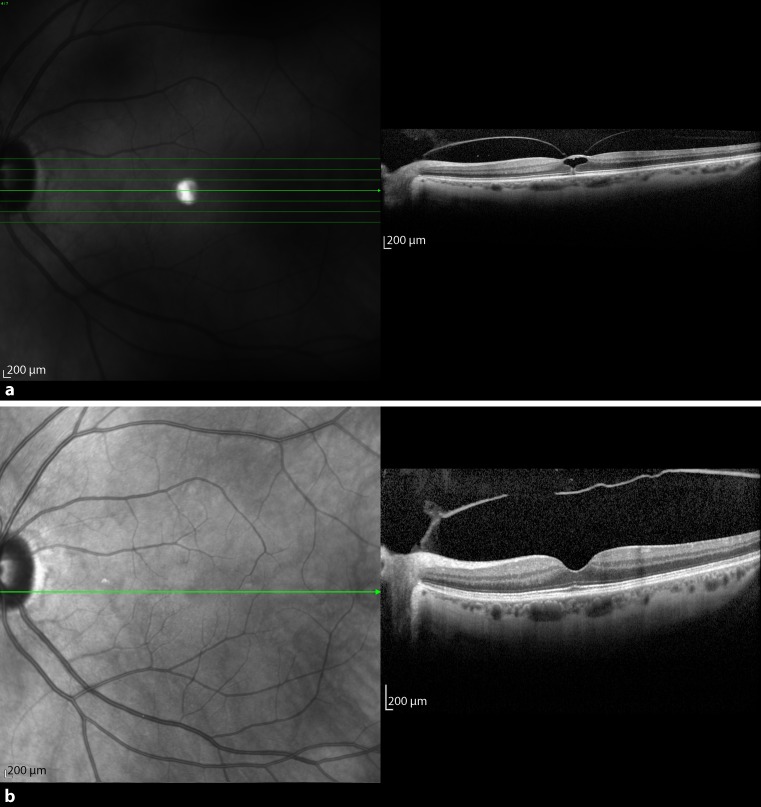
**a** Baseline optical coherence tomography (OCT) of the left eye of patient 5 with vitreomacular traction (VMT) and with a huge cyst in the inner retinal layer and disruption of the ellipsoid zone. **b** About 5 months postoperatively, OCT of the left eye shows VMT release and restoration of the ellipsoid zone

The fifth patient was a 50-year-old woman who presented with metamorphopsia in her left eye in September 2013. She was a myopic patient. On SD-OCT a VMT with an adhesion diameter of 397 μm and a macular cyst were diagnosed in the left eye with the right eye not showing any pathology (Fig. 5a). She had an initial BCVA of 1.0 and the foveal thickness was 335 μm. An intravitreal C_3_F_8_ gas injection with 0.3 ml was carried out 3 weeks after diagnosis. There was VMT release 173 days after injection with a foveal thickness of 205 μm and a consistent BCVA. There was no macula cyst detectable on SD-OCT after resolution (Fig. 5b).

**Fig. 6:**
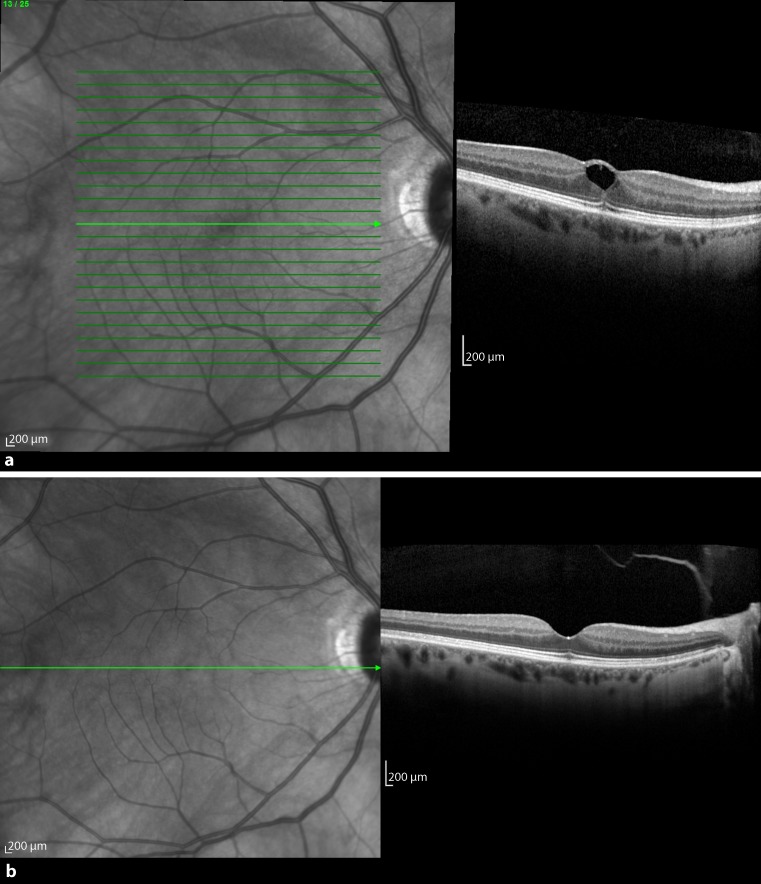
**a** About 1 year after the initial procedure, patient 5 presented with similar findings on optical coherence tomography (OCT) of the right eye. **b** Three weeks after intravitreal injection, OCT of the right eye shows vitreomacular traction release and restoration of the ellipsoid zone

In December 2014, when patient 5 was now aged 51, VMT with an adhesion diameter of 397 μm was diagnosed with SD-OCT in her right eye (Fig. 6a). At presentation, BCVA was 1.0 and foveal thickness was 351 μm. After 54 days of observation with a BCVA decrease on the right eye to 0.5, the patient received a 0.3-ml intravitreal C_3_F_8_ gas injection in her right eye. Three weeks later the vitreous body detached from the retina (Fig. 6b). After release, BCVA amounted to 1.0 again and foveal thickness was 204 μm.

### Patient 6

**Fig. 7:**
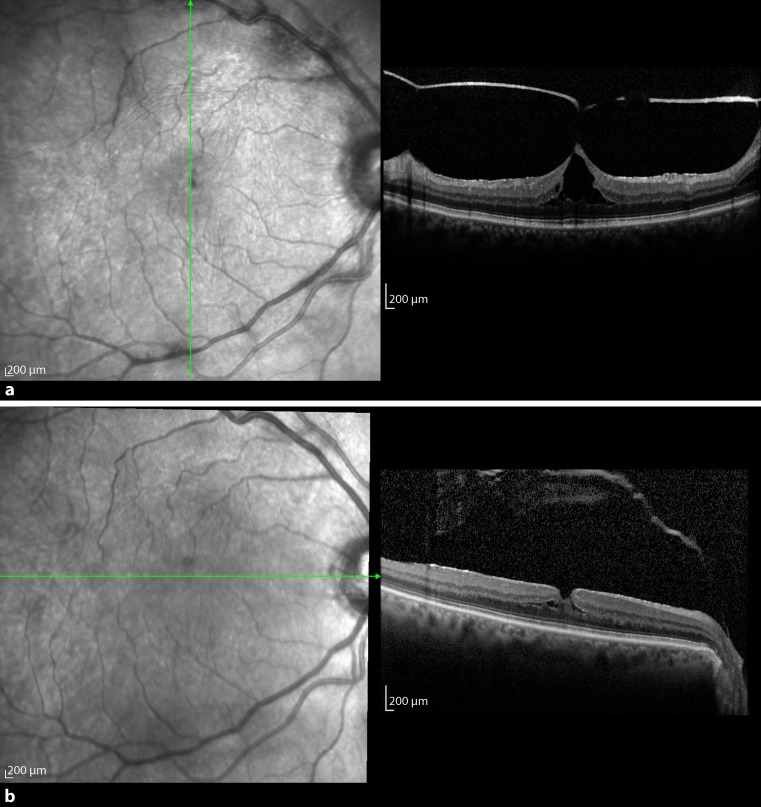
**a** Optical coherence tomography (OCT) of patient 6 obtained just before C_3_F_8_ intravitreal injection shows a tent-like elevation of the retina with intraretinal cystoid spaces due to vitreomacular traction force. **b** Five weeks after injection, there is resolution of vitreomacular traction with some residual intraretinal cysts on OCT

Patient 6 was a 75-year-old woman who was diagnosed with VMT in her right eye with OCT in September 2015 (Fig. 7a). At first presentation, the BCVA was 0.5. Additionally, the right eye showed a macula pucker. Foveal thickness and adhesion diameter measured 603 μm and 69 μm, respectively. Because no spontaneous resolution occurred after 47 days, the surgeon performed an intravitreal 0.2 ml 100% C_3_F_8_ injection. After 5 weeks, a separation of the vitreous from the macula could be seen on OCT (Fig. 7b). The BCVA of the patient did not change, but foveal thickness decreased to 270 μm.

## Discussion

This case series describes the use of a single intravitreal 100% C_3_F_8_ gas injection for the treatment of VMT with and without MH. This very small retrospective case series showed a successful release rate of VMT within the first month in 42.86% of patients and within 6 months in 85.71%. Only one patient (14.29%) failed to respond and had to undergo vitrectomy shortly after injection because of a retinal tear and detachment. The patient (14.29%) presenting with an MH showed VMT release, but had to undergo vitrectomy as a consequence of the nonclosure of the MH.

A very heterogeneous sample of patients was included, with patients showing concurrent ERM (42.86%) and diabetic retino- and maculopathy (14.26%). Nevertheless, successful release occurred with a very high frequency despite this patient population not being considered ideal for high success rates. Two patients (28.59%) experienced adverse events after the intravitreal gas injection, such as macular edema, retinal tear, and retinal detachment.

In this case series, the individual visual acuity improvements were modest. Visual acuity improved by two or more Snellen lines in 42.86% and remained stable in 57.14% of patients. It is disputable if and to what extent the BCVA of the patient with diabetic retino- and maculopathy had slightly decreased, regarding the fact that this diagnosis can cause severe loss of vision with time. Additionally, in one patient with a BCVA of 1.0, an increase in visual acuity could not be expected.

The two MIVI-TRUST studies, demonstrating the treatment efficacy of one single intravitreal 125-μg injection of ocriplasmin, showed that at day 28 postinjection, 26.5% and 40.6% of patients had resolution of adherence and nonsurgical MH closure, respectively. Both results were statistically significant [7]. The Ocriplasmin for Treatment for Symptomatic Vitreomacular Adhesion Including Macular Hole (OASIS) study reported even higher rates: 41.7% of patients in the ocriplasmin group had VMT release on day 28. Closure of MH without any surgical intervention at 3 months was reported in 30% of cases [8]. Sharma et al. found the resolution of VMT to be as high as 50% and nonsurgical closure of MH to be 27% in their sample [9]. All these studies only reported on the results of VMT resolution 28 days after the intravitreal injection and did not note release rates after 6 or 12 months, even though the OASIS trial had a 24-month follow-up. With a longer observation interval, it can be assumed that the results would also have increased. Moreover a long-term follow-up is favourable in order to assess the final visual acuity. Transient visual loss has been described in previous studies and a longer observation period can aid in definite judgment. In addition, further and larger “real-world” clinical series with heterogeneous patient collectives will offer more insight regarding patient groups, adverse events, and efficacy. There were numerous ocular exclusion criteria in the MIVI-TRUST study, which can hence hardly be compared with the patient collective of everyday life. Ocriplasmin is a first-in-class drug and its safety profile is nowhere near completion. The primary endpoint of the first and main studies was changes on OCT, although these do not always go hand in hand with visual improvement or decline and thus more studies are needed focusing also on visual gain [7, 8, 10].

Day et al. conducted a study with patients receiving an intravitreal sulfur hexafluoride injection for the treatment of VMT. A release rate of 55.6% as well as the closure of MH in two of two patients within the first month was documented, with the results being even better than with ocriplasmin. However, the study involved only nine patients compared with 464 treated with ocriplasmin in the MIVI-TRUST study, which makes these results hard to compare as it is harder to conclude about a population with fewer participants [7, 11]. Furthermore, the study did not include patients with ERM or other concurrent retinal diseases, which are according to Haller et al. favorable factors for pharmacologic release and hence could have increased their release rate [10, 11]. To date, only one study has been published with injection of sulfur hexafluoride (SF_6_) gas and further studies including patients with, for example, ERM are required to confirm these results.

Vitrectomy remains the gold standard for treatment of symptomatic VMT, showing release rates of up to 98%, nonetheless entailing the risk of intra- and postoperative complications [12]. It is dependent on a very experienced surgeon and requires patient suitability for surgery, which is not always the case in patients of advanced age. The main complication of cataract formation postoperatively is also worth considering, since this requires another surgery, if not automatically performed in the same intervention. Given the high success rate of vitrectomy, documented also after the completion of pharmacological therapy, it is possible in the future to perform vitrectomy as a second-line therapy only in cases where medical therapy has failed. With pharmacologic vitreolysis previous to vitrectomy, no additional side effects have been identified so far—on the contrary, Lopez-Lopez et al. even considered whether it could be used coadjuvantly by speeding up the surgery and minimizing its complications [3, 13].

It would be of high interest to directly compare the use of an intravitreal injection of ocriplasmin, C_3_F_8_ and SF_6_ gas, vitrectomy, and placebo for patients with VMT in a single clinical trial.

Rodrigues et al. were the first to investigate the use of an intravitreal injection of expansile C_3_F_8_ gas in treating VMT in 2013. Release of VMT was successfully observed in 40% of patients within the first month and increased to 60% within half a year [4]. In 2016, Steinle et al. assessed the posterior vitreous release rates after a single intravitreal injection of C_3_F_8_ for VMT treatment. At 1 month after injection, initial VMT release was 73% and increased to 83% by the final follow-up visit, which was on average 160 days [5]. The latest results in 2017 are from Chan et al., including 50 eyes, who reported on pneumatic vitreolysis with C_3_F_8_ in 86% of cases [6].

These studies show similar results of VMT release as those found at the Medical University of Graz. Rodrigues et al., on the one hand, included very few patients (15 eyes), while Chan et al., on the other hand, assessed 50 eyes, the largest collection of patients receiving a C_3_F_8_ gas injection to date. In all studies, including the case series performed in Graz, a very heterogeneous patient collective was included, with patients presenting with concurrent ERM, concurrent diabetes mellitus, exudative age-related macular degeneration, and MH. In the study conducted by Steinle et al., six eyes (20%) were included where previous intravitreal ocriplasmin injection had failed to release traction and one eye (3%) that had been included in another study receiving serial saline intravitreal injections. At the final follow-up, there was release in five of these six eyes (83%) and the eye with three intravitreal saline injections (one of one eye). This raises the question of whether a single C_3_F_8_ gas injection works more efficiently than ocriplasmin, and whether previous intravitreal injections aid in releasing traction or whether these adhesions would have perhaps released spontaneously.

Regarding ERM, Steinle et al. noted a success rate of 83% and suggested this therapy as a justifiable nonsurgical treatment in patients showing VMT and ERM [5]. In the case series of the Grazer Medical University, two out of three patients with concurrent ERM released traction, which is also a high rate but must be viewed critically owing to the small number of patients. However, these results are in contrast to those of Haller et al., who propose the absence of ERM as a positive predictive factor, which was associated with successful VMT release [10]. Rodrigues et al. also identified predictive factors that seem to have a positive impact on the traction release rate. All patients participating in the case series of the Medical University of Graz, who had a maximal horizontal VMA of lower than 750 μm (85.71%), showed a successful VMT release. These results strengthen the author’s assumptions. In the only patient who failed to respond, the extent of adhesion was 803 μm. The question arises of whether a larger adhesion diameter and thus a stronger tractive force are favorable for retinal tear and detachment, as was documented in this patient. However, the second postulated predictive feature of a maximal foveal thickness less than 500 μm cannot be seen in the case series of the Medical University of Graz. Three patients (42.86%) had a maximal foveal thickness of more than 500 μm, among whom two had VMT release within 6 months [4].

In none of the two studies was there a statistically significant improvement in mean visual acuity, which might be due to the relatively small number of included patients.

Successful closure of early-stage MH with gas injection was described as early as 1995 [14]. Rodrigues et al. included just one eye with an impeding MH at baseline, which progressed to a full thickness macular hole (FTMH) after C_3_F_8_ gas injection and eventually had to undergo vitrectomy for release of VMT. Steinle et al. had three eyes with FTMH of <200 μm at baseline, of which all three showed VMT release with gas and two had closure of their FTMH. The closure of stage 2 MH (<250 μm) was achieved, reported by Chan et al., in 66.7% of cases, while traction release was successful in all of these eyes. The Grazer case series notes one patient with an MH who had pneumatic release of VMT but required vitrectomy for closure of the persistent MH. It was previously described that MHs with a diameter of <200 μm have a higher chance of closure after medical therapy, which cannot be confirmed by the study done at the Medical University of Graz. The MH, however, did show a width of 201 μm and a single case is of very low significance [4, 5].

The main advantage of C_3_F_8_ gas is that it is easy to obtain, minimally invasive, and, last but not least, very cost-effective. Compared with a single injection of ocriplasmin, which costs about U.S. $ 3950, an intravitreal injection of C_3_F_8_ gas is much cheaper [15]. The choice of using C_3_F_8_ instead of SF_6_ is based on the longer intravitreal persistence and therefore the theoretically better effect on VMT.

### Limitations

The main limitation of this study is the number of patients, making it almost impossible to get statistically relevant results. Furthermore, the retrospective data collection and absence of a control group represented significant restrictions. The patient data were missing information such as the intraocular pressure before and after injection. Consequently, no statement can be made about these measures. In addition, the time from diagnosis to intravitreal C_3_F_8_ gas injection was very variable and the advised 3 months of observation was rarely observed. Moreover, the patient number is too small for evaluation of rare complications after intravitreal injection. Further studies addressing this very low-cost, minimally invasive, and promising treatment of symptomatic VMT are warranted.

## Conclusion

In conclusion, pneumatic vitreolysis with C_3_F_8_ gas is a promising nonsurgical option for treating VMT, with or without MH. The results of this case series suggest a further possibility of successful VMT release with a single intravitreal injection of C_3_F_8_ gas. Furthermore, C_3_F_8_ gas gives clinicians another option, besides ocriplasmin and SF_6_, to manage VMT in a less invasive manner. Additional studies with most notably a larger patient population are needed to further investigate the risk/benefit profile of C_3_F_8_ gas and to directly compare C_3_F_8_ gas with other methods of medical and surgical vitreolysis.
